# Statistical Downscaling for Rainfall Forecasts Using Modified Constructed Analog Method in Thailand

**DOI:** 10.1155/2017/1075868

**Published:** 2017-02-20

**Authors:** Patchalai Anuchaivong, Dusadee Sukawat, Anirut Luadsong

**Affiliations:** ^1^Department of Mathematics, Faculty of Science, King Mongkut's University of Technology Thonburi (KMUTT), 126 Pracha Uthit Road, Bang Mod, Thung Khru, Bangkok 10140, Thailand; ^2^The Joint Graduate School of Energy and Environment, King Mongkut's University of Technology Thonburi (KMUTT), 126 Pracha Uthit Road, Bang Mod, Thung Khru, Bangkok 10140, Thailand; ^3^Ratchaburi Learning Park, King Mongkut's University of Technology Thonburi (KMUTT), Rang Bua, Chom Bueng, Ratchaburi 70150, Thailand

## Abstract

The simulations of rainfall from historical data were created in this study by using statistical downscaling. Statistical downscaling techniques are based on a relationship between the variables that are solved by the General Circulation Models (GCMs) and the observed predictions. The Modified Constructed Analog Method (MCAM) is a technique in downscaling estimation, suitable for rainfall simulation accuracy using weather forecasting. In this research, the MCAM was used to calculate the Euclidean distance to obtain the number of analog days. Afterwards, a linear combination of 30 analog days is created with simulated rainfall data which are determined by the corresponding 5 days from the adjusted weights of the appropriate forecast day. This method is used to forecast the daily rainfall and was received from the Thai Meteorological Department (TMD) from the period during 1979 to 2010 at thirty stations. The experiment involved the use of rainfall forecast data that was combined with the historical data during the rainy season in 2010. The result showed that the MCAM gave the correlation value of 0.8 resulting in a reduced percentage error of 13.66%. The MCAM gave the value of 1094.10 mm which was the closest value to the observed precipitation of 1119.53 mm.

## 1. Introduction

It is difficult to predict the exact amount of precipitation in future events and prevent the likelihood of natural disasters. Henceforth, research and development of forecasting weather should be considered because rainfall is a crucial factor in sustaining life and the environment. Rainfall forecast plays an important role in maintaining water resources, the environment, and agriculture. Rainfall forecasts are still in the developing stages. They can be classified into 3 main methods [1–5]. The first method is statistical forecasting, based on finding the relationship between climatology data from past forecasts and future forecasts. This method is relatively simple but the relationship may suddenly change and it makes the forecasts less accurate. The second method is dynamical forecasting based on a climate model. This method requires a high-performance computer to generate sophisticated models and may also require large amounts of input data. The third method is hybrid forecasting which is based on the combination between statistical forecasting and dynamical forecasting which are applied together [1]. In general, this method provides a forecast with higher accuracy than the statistical method [2]. However, the resolution of the forecast is still too low for area-specific applications. A downscaling method is required. A downscaling method is a term used to explain the process of relating information or data with large-scale atmospheric variables that are provided by GCMs and reducing them to a finer, spatial, and temporal scale. In a more recent variety of articles, downscaling is widely used and applied in climatology for situations such as the construction, simulation, and prediction of the mean, minimum, and maximum air temperature and rainfall for the past 30 years [3]. Approaches for downscaling GCM simulations can be broadly classified as “dynamical” or “statistical” downscaling [4]. Dynamical downscaling is a technique that gathers output data from GCMs and uses that data to select a suitable regional and numerical model with a higher spatial resolution. This can simulate local climate conditions in greater detail. Techniques that employ regional climate models using fine grid spacing are quite efficient for forecasting [5–8]. Statistical downscaling techniques are based on a relationship between the larger scale climate predictors and observed precipitation. Predictors such as mean sea level pressure, humidity, geopotential height, relative humidity, and temperature may be used to downscale precipitation forecasts to the desired region and popularity [2–13]. There are a variety of methods for statistical downscaling [5], for example, the Delta Method (DM), Bias-Correction Method (BCM), Constructed Analogs Method (CAM), Localized Constructed Analogs Method (LOCA), Artificial Neural Networks (ANNs), Least Squares Support Vector Machines (LS-SVM), nonparametric kernel regression (NKR) [8–10], and so forth. In this research, the DM compares an arrangement of historical data and present day data with the actual records of measured data (monthly or daily observations) [14]. The BCM uses differences in observed climatology mean values between the GCM and observations from historical reference periods and is used to “correct” future GCM simulations [1]. BCSD is the GCM-simulated values that are “mapped” by quintile onto historical observed data. The AM uses data of weather forecasts in present day and records a day in the past when the weather scenario appears most similar (analog day) or finds an “appropriate match” analog for a forecast in the future [15]. The CAM uses a combination of analog days to forecast the temperature and improve the National Multimodel Ensemble's (NMME) method during the March-April-May (MAM) precipitation forecasts specifically used in studies at equatorial East Africa (EA) (by Shukla et al.) [16]. The area of study is between 2°S to 8°N and 36°E to 46°E. The results showed that precipitation and sea surface temperature (SST) forecasted over a large part of the Indo-Pacific Ocean (specifically between latitude 30°S to 30°N and longitude 30°E to 27°E, i.e., the analog domain) demonstrated high levels of absolute correlation with observed MAM precipitation over the EA (focus) region during the post-1999 period. Moreover, NMME closely resembles the precipitation forecasts over the analog domain and is used as a predictor for forecasting EA MAM precipitation. This generally provided higher levels of performance than when SST forecasts are used as predictors. Pierce et al. introduced a new technique (LOCA) for statistical downscaling simulations of daily temperature and precipitation [17] from using observations over the period from 1940 to 1969 when investigated. Observations between the periods 1970 to 2005 are used as testing data. They use anomalies when downscaling temperature and absolute values in precipitation. Results from downscaling the daily maximum temperature and precipitation illustrate that LOCA reproduces the extremes in summer maximum daily temperatures and winter daily precipitation quite well. A study found that many researchers have constructed predictions with experimental methods in a variety of ways that used statistical downscaling. So, statistical downscaling applications are preferable in the present day studies and are considered as one of the most cost-effective methods in local-impact estimates of climate scenarios and rainfall forecasts [18]. This method is of interest to developing countries and provides economic resources to streamline the recruitment system but requires high-performance computers. Climate change has significant impacts on human activity and natural disasters [3]. Thailand frequently faces large quantities of rain that causes the problem of flooding and damages the agriculture and affects industry and the people. These issues are the primary motivation for this research. The main objective of this study is to develop a rainfall forecast for Thailand using MCAM and compare observed precipitation at TMD stations where the investigation is carried out. The developed MCAM is designed to determine the appropriate measurement and coefficients in the linear combination. The Modified Constructed Analog Model is downscaled and provides estimates suitable for rainfall simulation accuracy using analog weather forecasting. Analog weather forecasting finds the best matching historical occurrence of a target pattern to determine an analog day with the MCAM for rainfall forecast at the station. Four predictors are used in each of the two datasets at the National Centers for Environmental Prediction (NCEP) Climate Forecast System Reanalysis (CFSR) and NCEP Climate Forecast System Version 2 (CFSv2) for the area covering Thailand. These predictors include the mean sea level pressure, temperature, moisture, and geopotential height at 850 hPa. Using the analysis field of CFSR (during the years between 1979 and 2009) and forecast field of CFSv2 (in the year 2010) and searching for the best matching historical pattern, it is possible to find analog day using the Euclidean distance formula. An analog day in the historical record (past data) will have the same characteristics as a predictor at a given target time. However, choosing a suitable predictor for the MCAM is important because it decreases the error in precipitation forecast based on the change in the coefficients. If the coefficient's value that is obtained from the CFSR and CFSv2 is too high, the forecasted precipitation error will also be high, respectively. The most suitable predictor must be selected from observing the lowest value Euclidean distance from similarity measurements. Once the analog day has been determined, the information can be used to forecast the precipitation of the current day (MCAM calibration). In order to test the performance of the method, it has been compared with the AM and the CAM. The AM and the CAM are the original methods that the MCAM is derived from. This paper is organized as follows.  Section 2 presents an overview of the study area and data used in this research, followed by methodology in Section 3. In Section 4, results of various analyses are presented and finally conclusions of the study are given in Section 5.

## 2. Data and Domain

In this research, experimental cases only select the predictors from the CFSR and CFSv2-Interim forecast dataset. These are the initial conditions for comparison between CFSR in years 1979 to 2009 (analysis data) and CFSv2 in the year 2010 (forecast data) [19–23]. The datasets of CFSR and CFSv2 have a variety of variables, but this research only shows the four variables that will be used as predictors: mean sea level pressure (MSLP), temperature (T850), moisture (Q850), and geopotential height (G850) at 850 hPa are used [2] (shown in Table 1). The data of the actual daily precipitation amount from the year 1979 to 2010 at the TMD are used for validation against the current year 2010.

### 2.1. Data for the Analysis Field

The National Centers for Environmental Prediction (NCEP) Climate Forecast System Reanalysis (CFSR) consists of 6-hourly time-series product from January 1979 to December 2010. The grid combines every six-hour forecast at 0000, 0600, 1200, and 1800 UTC per day and the resolution is 0.5 × 0.5 degrees latitude-longitude [19–21].

### 2.2. Data for the Forecast Field

The NCEP Climate Forecast System Version 2 (CFSv2) data are 6-hourly produced from the National Centers for Environmental Prediction (NCEP) Climate Forecast System (CFS), which is initialized four times per day (0000, 0600, 1200, and 1800 UTC). Every 5 days, 6-hourly atmospheric, oceanic, and land surface analyzed products and month forecasts are available at 1-degree latitude-longitude horizontal resolution and are then interpolated into a 0.5-by-0.5 grid. From this dataset, the data has been downloaded by using the starting date on 1 January 2010 [22, 23].

### 2.3. Data for of the Actual Daily Precipitation

The actual daily precipitation used in the calculations during 15 May to 15 October throughout the years 1979 to 2010 at the TMD stations is used for validation against the current year 2010.

**Figure 1:**
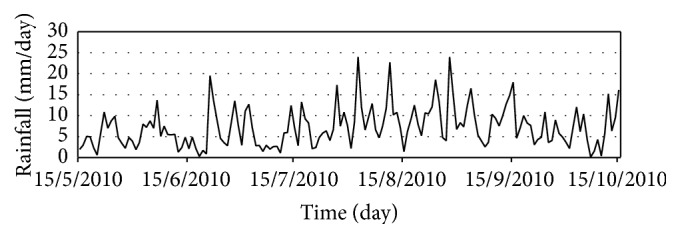
Mean rainfall (mm/day) during 15 May to 15 October 2010 at the 30 stations.

In the standard analysis for the daily rainfall in Thailand, the past rainfall data during the years of 1979 to 2010 were recorded with measurement tools such as the rain gauge. The rain gauge measures the height of precipitation that falls onto a set area in millimeters. There are two types of rain gauges: the nonrecording rain gauge and the recording rain gauge. There are a total of 80 meteorological stations in Thailand that record this data daily from 7.00 a.m. to 7.00 a.m. of the next day. However, in these 80 stations, there are missing data. Therefore, to ensure that this research is accurate, only 30 out of the 80 stations which do not have missing data were selected.  Figure 1 shows the mean rainfall (mm/day) during 15 May to 15 October 2010 at the 30 stations.

### 2.4. Domain

**Figure 2:**
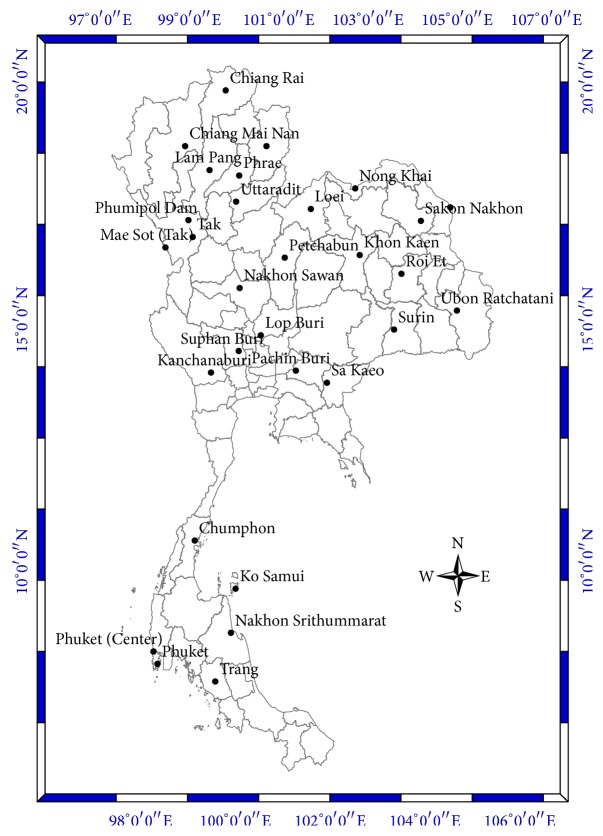
Location map of the study area in Thailand.

The model domain of CFSR and CFSv2 covers the area between latitude 90°S to 90°N and longitude 180°W to 180°E. The study domain only covers the areas of Thailand between latitude 4°N to 22°N and longitude 95°E to 110°E as shown in Figure 2. The locations of the meteorological stations in Thailand for downscaling at the 30 stations (1979–2010) are divided into five regions: north, northeast, central, western, and south. These locations of the thirty meteorological stations that have been used in the experiments are shown in Table 2 and Figure 1.

## 3. Methodology

Similarity measure is a function which computes the degree of similarity between a pair of objects. Similarity measure can be done in a variety of ways such as using Euclidean distance and absolute error [24]. However, only the Euclidean distance has been developed as a calculation method. The Euclidean distance is the shortest distance between two points, which is a line [14]. Euclidean distance between *P*_*i*_ and *Q*_*i*_ is defined by

(1) $$\begin{array}{c} D_{EU}={\left(\;{\sum_{i=1}^{n}\left(P_{i}-Q_{i}\right)}^{2}\;\right)}^{1/2}. \end{array}$$

Therefore, distance measurements have been applied by searching for the day in history most similar to the forecast day. Euclidean distance can be applied in many ways such as the AM and the CAM. The MCAM can estimate the rainfall forecast (mm/h) at the station. For example, the AM, the CAM, and the MCAM are represented here.

### 3.1. Analog Method (AM)

The analog method is a simple statistical downscaling method which is based on the selection of similar atmospheric states. The performance of the AM is dependent on the degree of similarity. Wetterhall et al. [25] described that the basic idea of AM is to find a predictor from the historical record which has the same characteristics as a predictor at a given target time.

Let *G*(*t*) be predictors from the GCM:

(2) $$\begin{array}{c} G\left(\;t\;\right)=\left[\;G_{1}\left(\;t\;\right),\ldots ,G_{L}\left(\;t\;\right)\;\right]. \end{array}$$

Let *A*(*t*) be predictors from observation (analysis data):

(3) $$\begin{array}{c} A\left(\;t\;\right)=\left[\;A_{1}\left(\;t\;\right),\ldots ,A_{n}\left(\;t\;\right)\;\right]. \end{array}$$

Therefore, Euclidian distance for the analog method to find analog day is defined as in

(4) $$\begin{array}{c} D={\left(\;\sum_{n=1}^{N}{\left[\;G_{n}\left(\;t\;\right)-A_{n}\left(\;t\;\right)\;\right]}^{2}\;\right)}^{1/2}, \end{array}$$

where *G*_*n*_(*t*) is forecast predictor during 15 May to 15 October 2010 (forecast data, d). *A*_*n*_(*t*) is a predictor from observation during 15 May to 15 October between the years 1979 and 2009 (analysis data). *N* is the number of grid points (*n* = 1,…, *N*).

The analog days of the analog method for each forecast day are determined from the corresponding 1 day of analysis data. For example, in the case of 15 May 2010 forecast, the analog method is determined by comparing forecast of 15 May, with the past data of 15 May of the years 1979 to 2009. The analog day is calculated by Euclidean distance in comparing the year. For the case of 15 May forecast, we got 31 analog days, but we will choose the minimum value compared with the Euclidean distance in each year. Then, we get an analog day for daily measurement (rainfall forecast/time).

### 3.2. Constructed Analog Method (CAM)

The constructed analog is a technique in statistical downscaling which is inspired by analog weather forecasting [16]. The difference between constructed analog and analog method is that the constructed analog creates the analog from a linear combination of 30 analog days. By measure of similarity of analog for two anomalies, “maps” observed at *t*_*i*_ and *t*_*j*_ consist of the following two expressions.

#### 3.2.1. Root Mean Squared Difference (RMSD)

This is defined as

(5) $$\begin{array}{c} \text{RMSD}={\left(\;\sum_{i=1}^{N}{\left\{\;f\left(\;s,t_{i}\;\right)-f\left(\;s,t_{j}\;\right)\;\right\}}^{2}\;\right)}^{1/2}, \end{array}$$

where *f*(*s*, *t*_*i*_) is forecast predictor during 15 May 2010 (forecast data), *f*(*s*, *t*_*j*_) is a predictor from observation during 15 May between the years 1979 and 2009 (analysis data), and *n* = 1,…, *N* (number of grid points) [26].

The CAM is applied to determine the analog days for each forecast day and is determined from the corresponding 1 day of analysis data. To determine of coefficients for the linear combination is the main concept of the CAM.

#### 3.2.2. Linear Combination for the CAM

Given an initial condition *f*^IC^(*s*, *j*_0_, *m*), for example, the most recent state (monthly mean map), where *j*_0_ is outside the range *j* = 1,…, *M*, suitable monthly climatology is removed from the data; henceforth, *f* shall be the anomaly [27]. A constructed analog is defined as

(6) $$\begin{array}{c} f^{CA}\left(\;s,j_{0},m\;\right)=\overset{M}{\underset{j=1}{\sum }}\alpha_{j}f\left(\;s,j,m\;\right), \end{array}$$

where *m* is month (*m*), *j*_0_ is outside the range *j* = 1,…, *M*, *M* is year (*j* = 1,…, *M*), and *α*_*i*_ are coefficients to be determined to minimize the difference between *f*^CA^(*s*, *j*_0_, *m*) and *f*^IC^(*s*, *j*_0_, *m*). The technical solution to this problem is discussed below in (5) and involves manipulating the alternative covariance matrix *Q*^*a*^. An approximated solution to this problem is given by Van Den Dool [26]. In this study, rainfall forecast for the CAM coefficient is 0.1 [28].

### 3.3. Modified Constructed Analog Method (MCAM)

Modified Constructed Analog Methods are developed from the CAM [27] and the AM [25] with two steps using a technique in statistical downscaling which is inspired by analog weather forecasting. There are 2 steps to develop the MCAM, by determining the appropriate measure in (7) and determining the appropriate method for finding coefficients in the linear combination in (11).

#### 3.3.1. Appropriate Measure of Modified Constructed Analog Method

The Euclidian distance for Modified Constructed Analog Method is defined as follows:

(7) $$\begin{array}{c} {\text{EU}}_{\text{MCAM}}={\text{MW}}_{\alpha_{i}}\sqrt{C}. \end{array}$$

Hence,

(8) $$\begin{array}{c} C=\overset{N}{\underset{n=1}{\sum }}{\left(\;f^{\text{PF}}\left(\;s,j,m,t,d\;\right)-f^{\text{AF}}\left(\;s,j_{0},m,t,d\;\right)\;\right)}^{2}, \end{array}$$

where *f*^PF^(*s*, *j*, *m*, *t*, *d*) is the forecast predictor during 15 May to 15 October 2010 (forecast data). *f*^AF^(*s*, *j*_0_, *m*, *t*, *d*) is a predictor from observation during 15 May to 15 October between the years 1979 and 2009 (analysis data) determined from the corresponding 5 days. MW_*α*_*i*__ is the weight vector by a nonnegative real number and *α*_*i*_ are the coefficients to be determined so as to minimize the difference between *f*^PF^(*s*, *j*, *m*, *t*, *d*) and *f*^AF^(*s*, *j*_0_, *m*, *t*, *d*) at node *i* at iteration *n* (*n* = 1,…, *N*) (number of grid points). For determining the corresponding 5 days in comparison, we will get an analog day as 155 days/time. Selection of 30 analog days with the minimum Euclidean distance comes from the calculation of 1979–2009 which is similar to the previous year, 2010. Then, we can determine the rainfall forecast at the monitoring stations according to the principle of downscaling techniques. Determining the coefficients for the linear combination is the main concept of the MCAM. To summarize, the concept of the method is to form the best matching historical occurrence of a target pattern and it is assumed that the weather will evolve the same way it did before. The MCAM is a method used to find and select a suitable analog day from a linear combination of the best 30 analog days. Reducing errors in forecasting rainfall by experimentation to find the appropriate method and their coefficients in the linear combination in equations will be presented in the next section.

#### 3.3.2. Finding the Weight of the Modified Constructed Analog Method

The weight of the Modified Constructed Analog Method based on the weighted sum method by solution to the problem presented in (7) is MW_*i*_ if the weight is positive for all. The updated new value of the weight at iteration *i* can be written as

(9) $$\begin{array}{c} \frac{{\text{MIMEU}}_{\text{MCAM}}}{\sum_{i=1}^{m}{\text{MIMEU}}_{\text{MCAM}}}={\text{MW}}_{i}, \end{array}$$

where MIMEU_MCAM_ is the smallest Euclidean distance that was selected in 30 analog days from a total of 155 analog days with predictor data and *m* is the number of analog days (*m* = 1,…, 30). By weight, the sum can be defined as

(10) $$\begin{array}{c} \overset{n}{\underset{i=1}{\sum }}{\text{MW}}_{i}=1, \end{array}$$

where *n* is the number of weights (*n* = 1,…, 30). The weight of a nonnegative real number is obtained with actual data in each forecast from the calculation.

#### 3.3.3. Linear Combination Method for MCAM

The linear combination method is defined as follows:

(11) $$\begin{array}{c} V_{s}={\text{MW}}_{1}{\text{RF}}_{1}+{\text{MW}}_{2}{\text{RF}}_{2}+\cdots +{\text{MW}}_{30}{\text{RF}}_{30}, \end{array}$$

where *V*_*s*_ is the value daily rainfall forecast of predictors (G850, MSLP, Q850, and T850) (mm/h) (calibration). RF_*i*_ is the observed rainfall at the stations (analog day). MW_*i*_ is the weighted data (*i* = 1,…, 30).

#### 3.3.4. The Value Rainfall Forecast in Each Predictor for MCAM

By linear combination for the Modified Constructed Analog Method, the updated predictor data used for daily rainfall forecast in the Modified Constructed Analog Method is defined as

(12) RFG850=w1,g850g1,g850+w2,g850g2,g850+⋯+wk,g850gk,g850,RFMSLP=w1,mslpm1,mslp+w2,mslpm2,mslp+⋯+wk,mslpmk,mslp,RFQ850=w1,q850q1,q850+w2,q850q2,q850+⋯+wk,q850qk,q850,RFT850=w1,t850t1,t850+w2,t850t2,t850+⋯+wk,t850tk,t850,

where *w*_*k*_ is the weight, *g*_*k*,g850_ is the daily forecasted precipitation value for G850 (mm/h), *m*_*k*,mslp_ is the daily forecasted precipitation value for MSLP (mm/h), *q*_*k*,q850_ is the daily forecasted precipitation value for Q850 (mm/h), and finally *t*_*k*,t850_ is the daily forecasted precipitation value for T850 (mm/h) observed at the stations (*k* = 1,…, 30).

### 3.4. The Average of Rainfall Forecast for Four Predictors in the AM, the CAM, and the MCAM

This method is a simple and precise method for calculating and forecasting regional rainfall volume [5]. The new updated value for the average of AM, CAM, and MCAM in 0000, 0600, 1200, and 1800 UTC can be written as in

(13) Average AM=0.25SRFG850+SRFMSLP+SRFQ850+SRFT850,Average CAM=0.25SRFG850+SRFMSLP+SRFQ850+SRFT850,Average MCAM=0.25SRFG850+SRFMSLP+SRFQ850+SRFT850,

where SRF_*p*_ is the observed rainfall forecast (mm/h) (*p* is a predictor) (see (13)), with the rainfall forecast for all predictors (four times). Creating the situations of a consistent spatial pattern of rainfall at the stations is required.

### 3.5. Performance Criteria for Rainfall Forecast

To evaluate the performance of each of the three indexes, the prediction error can be calculated: the correlation coefficient (*R*^2^), the root mean square error (RMSE), and the mean absolute percentage error (MAPE) [26].

#### 3.5.1. Correlation Coefficient (*R*^2^)

The coefficient (*R*^2^) is a measure of linear correlation of two variables. It indicates how well the observation and forecast value fit a line. This can be estimated by [1]

(14) r=n∑s=1nRFsOBSs−∑s=1nRFs∑s=1nOBSsn∑s=1nRFs2−∑s=1nRFs2n∑s=1nOBSs2−∑s=1nOBSs2,R2=1−∑s=1nRFs−OBSs2∑s=1nOBSs−OBS¯2,

where OBS_*s*_ is the value observed at station in Thailand (actual value), RF_*s*_ is the rainfall forecast of predictors (forecast value), and $\bar{OBS}$ is the mean values of OBS_*s*_ (observed rainfall).

To determine the level of correlation, a coefficient in the range between −1 and +1 is used. The sign shows the direction of correlation. When *r* is close to −1 or +1, this indicates a high level of correlation. When *r* is 0 or close to 0, this indicates little or no correlation. Shown in Table 3 are the levels of correlation.

#### 3.5.2. Root Mean Square Error (RMSE)

The RMSE is frequently used to indicate the sample standard deviation of the forecast and observation, defined as follows [1]:

(15) $$\begin{array}{c} \text{RMSE}=\sqrt{\frac{\sum_{s=1}^{n}{\left({\text{OBS}}_{s}-{\text{RF}}_{s}\right)}^{2}}{n}}. \end{array}$$

#### 3.5.3. The Mean Absolute Percentage Error (MAPE)

The mean absolute percentage error is a measure of accuracy of the method for constructing rainfall forecasting of the predictors at the station number in statistics, specifically in trend estimation. It usually expresses accuracy as a percentage and is defined by the following equation:

(16) $$\begin{array}{c} \text{MAPE}=\frac{1}{n}\overset{n}{\underset{s=1}{\sum }}\left(\;\left|\;\frac{{\text{OBS}}_{s}-{RF}_{s}}{{\text{OBS}}_{s}}\;\right|\times 100\;\right), \end{array}$$

where OBS_*s*_ is the value of observed rainfall at the stations in Thailand (actual value) and RF_*s*_ is the rainfall forecast of predictors (forecast value). The closer the values of correlation coefficient are to 1, the more accurate the data will be. Simulations are considered satisfactory when MAPE is below 10% and excellent when MAPE is less than 5% [8]. A percentage error of 0 indicates that the forecasted rainfall and the actual observed rainfall are identical.

### 3.6. Experiments

In this research, rainfall forecasts by using AM, CAM, and MCAM are conducted. Comparisons between the averages from all predictors of rainfall forecast (mm) and observed rainfall at the stations during 15 May to 15 October in 2010 are investigated. Accuracy is investigated in the method along with the percentage error. The experiment cases are shown in Table 4.

**Figure 3:**
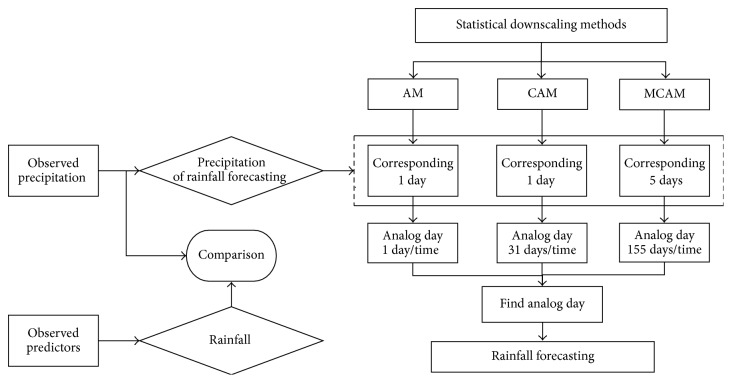
The process for the AM, CAM, and MCAM in this study.

The process for the AM, the CAM, and the MCAM of the research in this paper is described in Figure 3.

**Figure 4:**
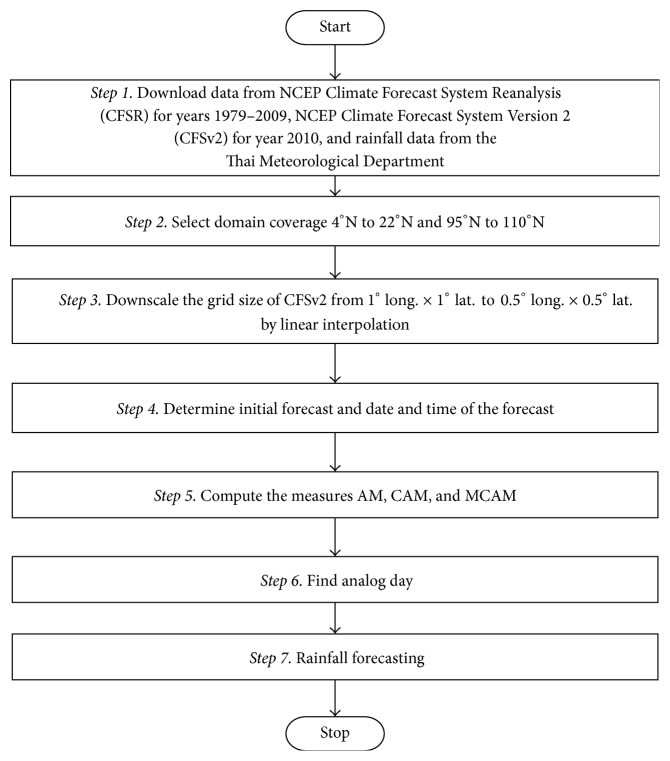
Flow chart showing the steps of simulation for AM, CAM, and MCAM at 0000, 0600, 1200, and 1800 UTC.

The steps for the simulation of AM, CAM, and MCAM are shown in Figure 4.

#### 3.6.1. Steps for the AM, the CAM, and the MCAM

**Step 1 .**

**Figure 5:**
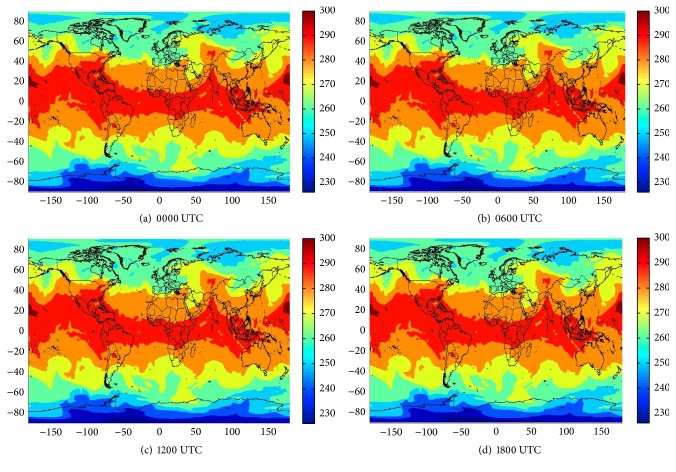
Data from CFSv2 of year 2010 (15/05/2010). Predictors: T850.

Download data from the NCEP Climate Forecast System Reanalysis (CFSR) for years 1979 to 2009, NCEP Climate Forecast System Version 2 (CFSv2) for year 2010, and rainfall data from the Thai Meteorological Department. Examples of this data are shown in Figure 5.

**Step 2 .**

**Figure 6:**
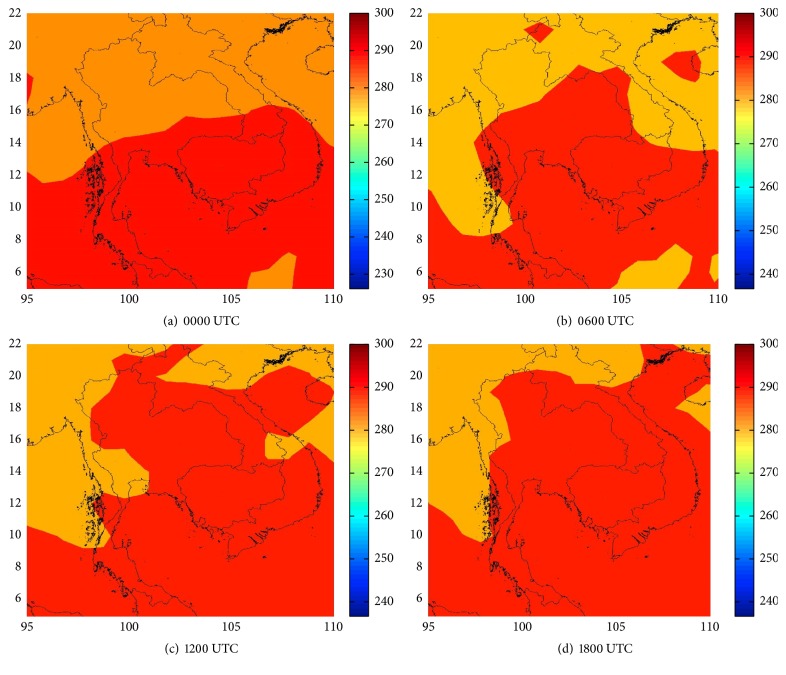
Cropped image of CFSv2 (15/05/2010). Predictors: T850.

Select the domain coverage from 4°N to 22°N and 95°E to 110°E in Thailand. Examples of this data are shown in Figure 6.

**Step 3 .**

Downscale the grid size of CFSv2 from 1° long. × 1° lat. to 0.5° long. × 0.5° lat. by linear interpolation.

**Step 4 .**

Determine the initial forecast, date, and time in Table 4.

**Step 5 .**

Compute the measurement to find the analog day of the AM using (4), the CAM using (5), and the MCAM using (7).

**Step 6 .**

Find the analog day.

**Step 7 .**

Forecast daily rainfall value based on the analog day.

## 4. Results and Discussion

**Figure 7:**
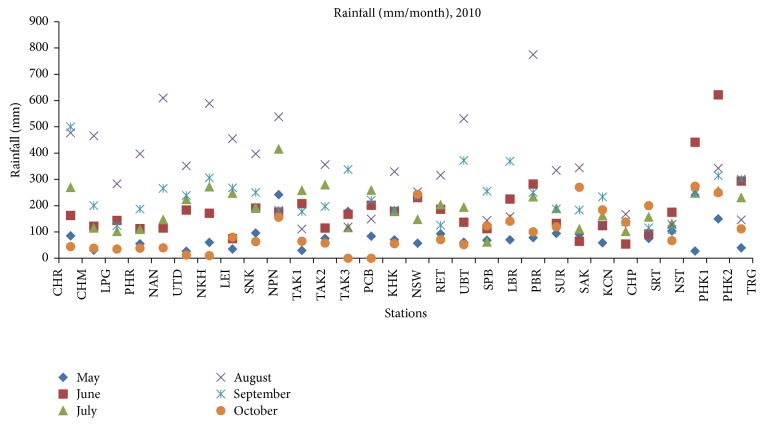
Rainfall (mm/month) at the 30 stations.

In this research, the results for forecasting rainfall from 15 May to 15 October in the year 2010 using AM, CAM, and MCAM are compared. The total area is approximately 513,120 km^2^. Overall, Thailand has a humid subtropical climate with fairly high precipitation. The mean annual rainfall (during 15 May to 15 October 2010) is 1119.53 mm and average rainfall (during 15 May to 15 October throughout the years 1979–2009) is 1066.8 mm measured at 30 stations (shown in Figure 7) [29, 30]. The data demonstrated that the amount of precipitation has increased due to a low pressure trough and a southwestern monsoon that arrives to cover Thailand during the rainy season. The low pressure trough that passes across the country causes precipitation starting from the beginning of the rainy season throughout the months of May and July. In July, the low pressure will shift south again and causes continuous heavy rains until the northwestern monsoons arrive to cover Thailand. When the southwestern monsoon comes to replace the northwestern monsoon during mid-August, northern Thailand will start to have cold weather and decreased rainfall. However, the south will still continue to experience heavy rains. This information is in accordance with the observed rainfall at the meteorology station.

**Figure 8:**
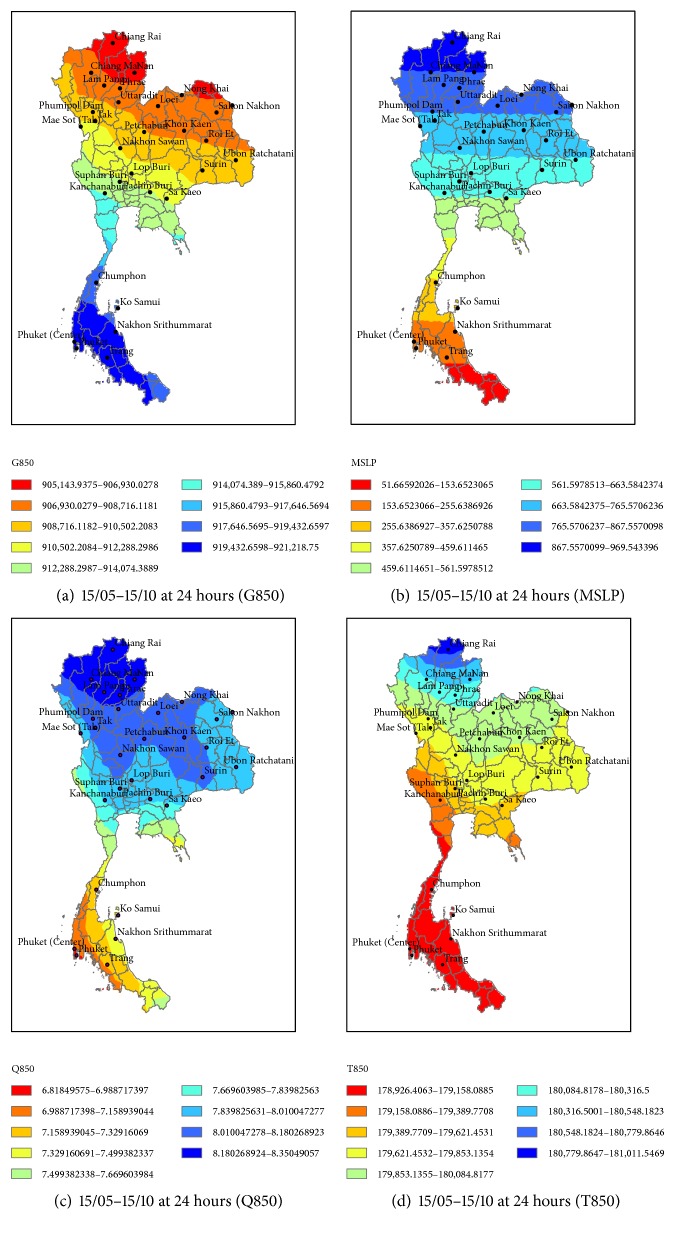
The 850 hPa geopotential height (G850), mean sea level pressure (MSLP), 850 hPa moisture (Q850), and 850 hPa temperature (T850) from CFSv2 during 15 May to 15 October 2010 at the 30 stations in Thailand.

**Figure 9:**
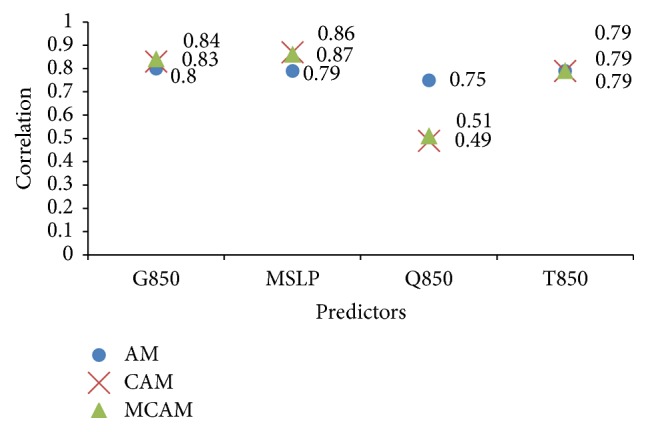
The histogram of the correlation between observations and simulations for four predictors, represented by mean sea level pressure, temperature, moisture, and geopotential height at 850 hPa of the AM, the CAM, and the MCAM decomposition.

The following intervals are used to consider the amount of daily precipitation: very dry at >0.1 mm, normal at 10.1–35 mm, and very wet at <90.1 mm [27]. The performance of the forecast predictor during 15 May to 15 October 2010 (forecast data) in Thailand is shown in Figure 8. To determine the performance of each predictor, the RSME and MAPE can be checked [26]. The analyzed correlation between the observed and simulated rainfall is shown in Tables 5 and 7. These are the results from the experiment of rainfall forecast between observed and simulated rainfall by the AM, the CAM, and the MCAM taking into account atmospheric predictors over Thailand regions at MSLP, T850, Q850, and G850. The forecast predictor during 15 May to 15 October 2010 at 30 stations in Thailand is based on statistical downscaling. The actual rainfall and forecasted rainfall are displayed in Tables 5–7. From Table 5, by using the two methods, the four predictors G850, MSLP, Q850, and T850 had a positive correlation overall. The values vary according to the amount of rainfall. When the amount of actual observed rainfall increases, the amount of predicted rainfall also increases accordingly. The results for the AM predictors gave the following correlation values: G850 gave 0.8, MSLP gave 0.79, Q850 gave 0.75, and T850 gave 0.79. The CAM predictors gave the following correlation values: G850 gave 0.83, MSLP gave 0.87, Q850 gave 0.49, and T850 gave 0.79. Between the two methods, CAM gave the G850 predictor with the highest correlation value at 0.83 and the lowest correlation value with Q850 at 0.49. The average forecasted rainfall was summed and compared with the average actual observed rainfall for all 30 TMD stations in Thailand. The AM method displayed the closest similarity for all predictors to the actual observed rainfall. Finally, the MCAM predictors gave the following correlation values: G850 gave 0.84, MSLP gave 0.86, Q850 gave 0.51, and T850 gave 0.79. MCAM gave the MSLP predictor with the highest correlation value at 0.86 and the lowest correlation value with G850 at 0.51 as shown in Table 6. However, these three methods gave the various correlations which are acceptable to statistical calculations and shown in Figure 9.

Tables 5 and 6 can be summarized as the value of average rainfall between observed and simulated rainfall for all four predictors. It is found that the average rainfall (observed) at 30 stations is 1119.53 mm. AM gave average rainfall similar to the observed rainfall at Q850 which was 1091.04 mm and the percentage error was 2.54%. CAM gave average rainfall differing from the observed one with high percentage error. MCAM give average rainfall similar to the observed one in T850 at 1133.12 mm and the percentage error is 1.2%.

**Figure 10:**
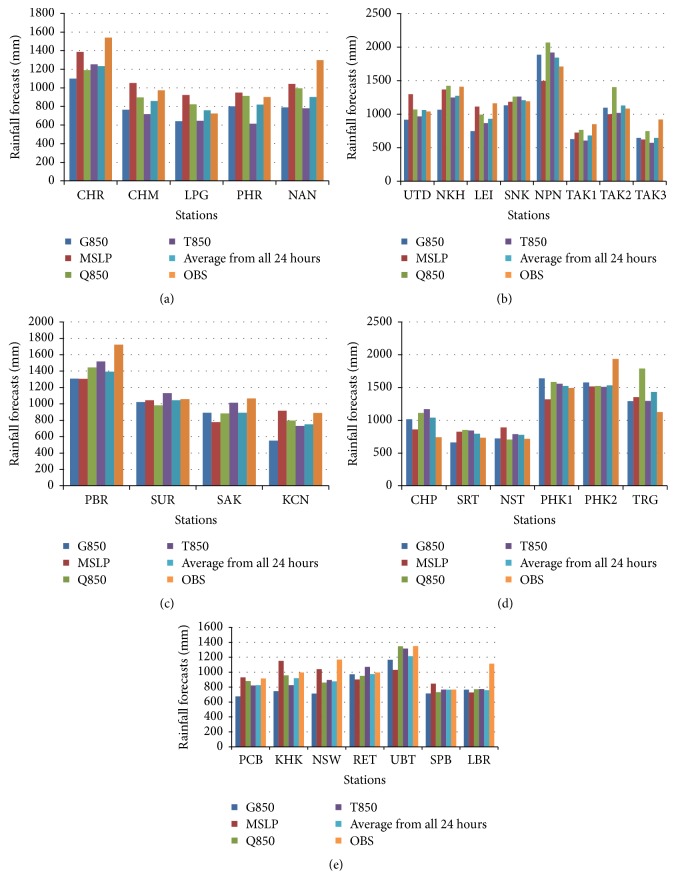
The histogram of the comparison between observed stations and simulations for four predictors, represented by an average from all 24 hours, mean sea level pressure, temperature, moisture, and geopotential height at 850 hPa of AM decomposition. This study shows five provinces: (a) northern, (b) northeast, (c) western, (d) southern, and (e) central.

**Figure 11:**
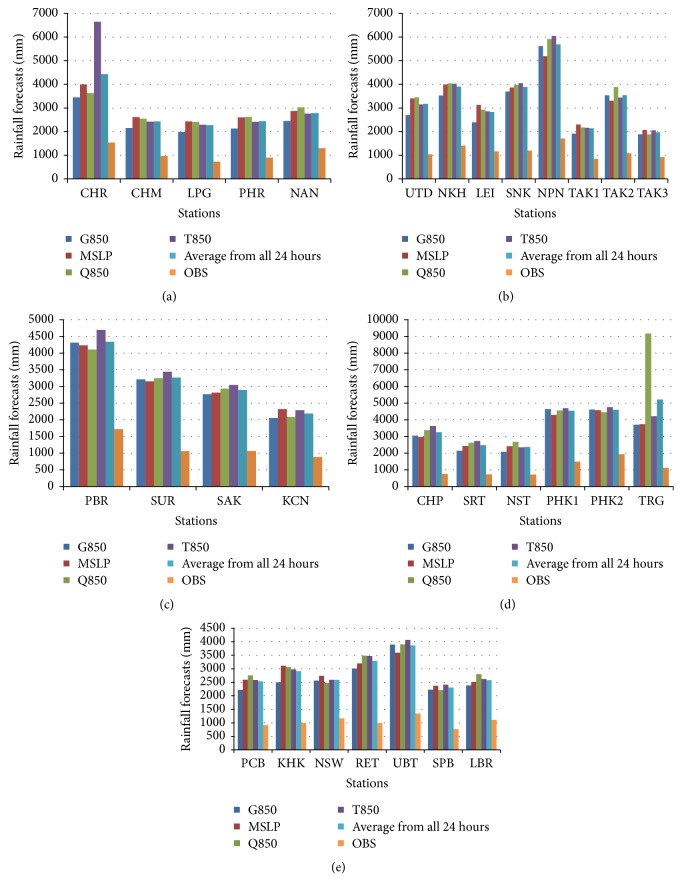
The histogram of the comparison between observed stations and simulations for four predictors, represented by an average from all 24 hours, mean sea level pressure, temperature, moisture, and geopotential height at 850 hPa of CAM decomposition. This study shows five provinces: (a) northern, (b) northeast, (c) western, (d) southern, and (e) central.

**Figure 12:**
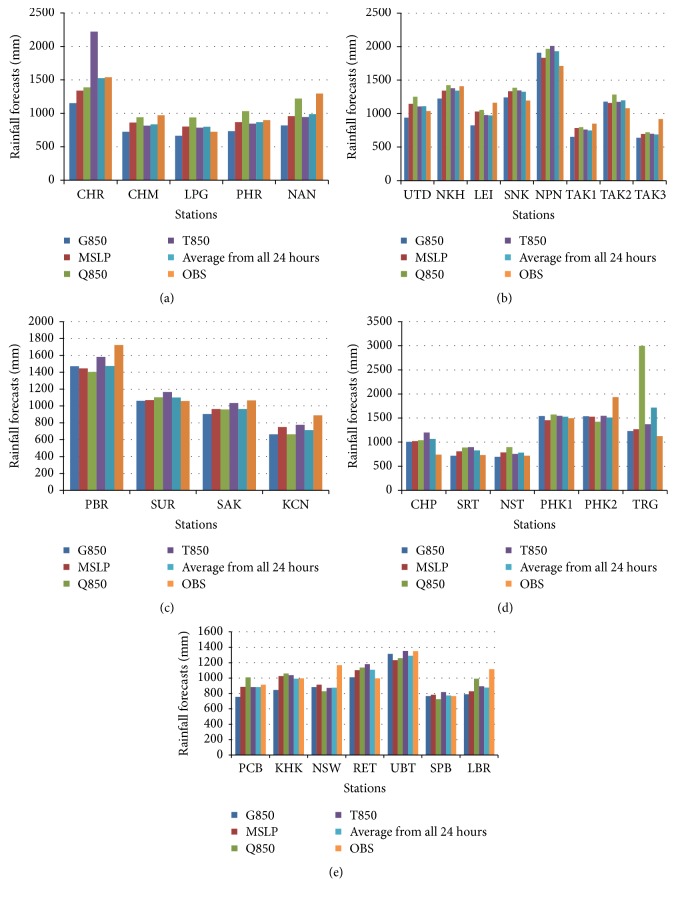
The histogram of the comparison between observed stations and simulations for four predictors, represented by an average from all 24 hours, mean sea level pressure, temperature, moisture, and geopotential height at 850 hPa of MCAM decomposition. This study shows five provinces: (a) northern, (b) northeast, (c) western, (d) southern, and (e) central.

The results pointed out that MCAM gave a result most similar to the optimized forecast with the least amount of percentage error out of the three methods. This research displays data for the observed rainfall and simulated rainfall using the four predictors which are divided into five regions in Thailand. The data is identified in histogram graphs (Figures 10–12).

**Figure 13:**
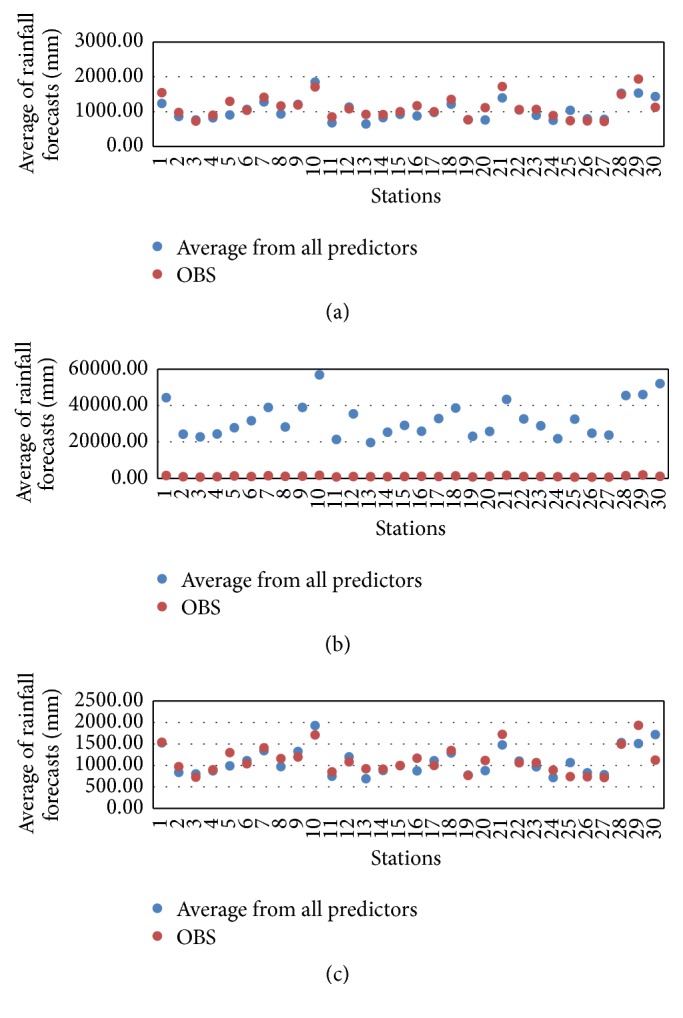
Comparison between averages from all predictors of rainfall forecast (mm) (horizontal axis) and stations (vertical) of (a) AM, (b) CAM, and (c) MCAM. For averages rainfall forecast during 15 May to 15 October 2010 at 30 stations in Thailand.

**Figure 14:**
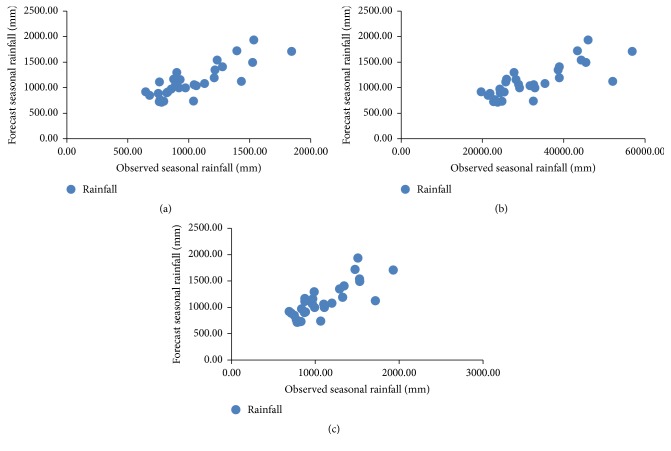
Scatter plot of observed (horizontal axis) and forecast (vertical) rainfall for (a) AM, (b) CAM, and (c) MCAM.

**Figure 15:**
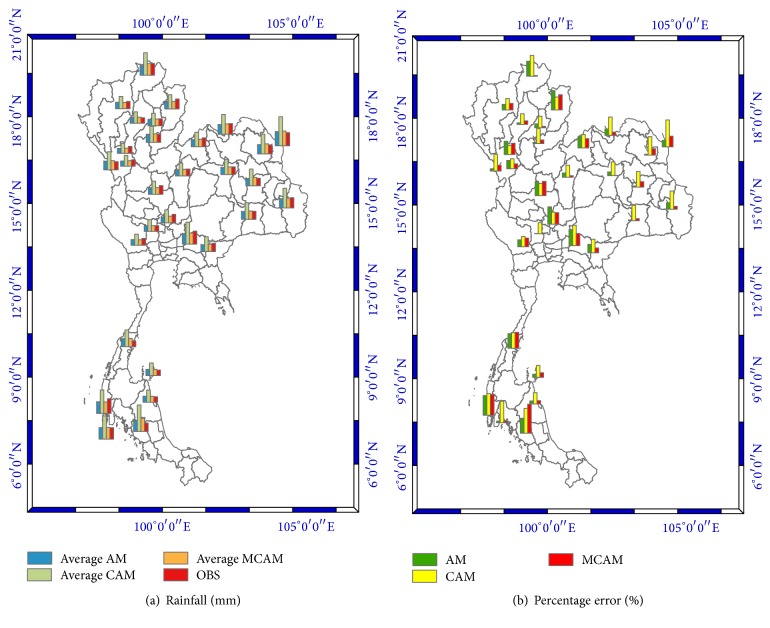
(a) shows the bar graphs for rainfall in millimeters as follows: the average forecasted rainfall by using the AM (blue), the average forecasted rainfall by using the CAM (green), the average forecasted rainfall by using the MCAM (orange), and the actual observed rainfall (red). (b) shows the bar graphs of percentage errors for each of the methods as follows: AM in green, CAM in yellow, and MCAM in red.

These figures show that the forecast percentage errors in the three methods are different but the MCAM gave the rainfall forecast which is most similar to the observed rainfall at NKH, SUR, SAK, PCB, SPB, SRT, NST, and PHK1 which is satisfactory. Performances of the rainfall forecast between observed and simulated rainfall for all predictors are shown and summarized in Table 7 (Figures 13–15).

Performance of the forecast predictor during 15 May to 15 October 2010 (forecast data) is shown in Figure 8.

Another point of interest is the correlation between observed and average rainfall from all predictors in Table 7 (Figures 10–12), which is higher than 0.82 (*R*^2^ = 0.67) using AM, and the lowest performance correlation is 0.79 (*R*^2^ = 0.61) using CAM. AM gave more correlation than CAM and MCAM, but MCAM gave the minimum percentage error (13.66%). The experimental results are summarized in Table 7 and are compared with the results in Figure 13. This is another way that the application of statistical downscaling can be used for rainfall forecasting by using the MCAM in Thailand.

## 5. Conclusion

This paper introduces another method for the development of rainfall forecasting in Thailand. The MCAM is used for statistical downscaling with the four predictors (T850, G850, Q850, and MSLP) when the amount of precipitation is being compared at the stations. Hence, the present downscaling approach is suitable for the simulation of rainfall under changed climate from GCMs [10]. The MCAM investigates rainfall forecasting in five regions at 30 stations in Thailand. This method is compared with AM and CAM. It can reduce the problem of errors in forecasts, the need of intensive computational resources, and the management of large data while simplifying output data. The MCAM is linearly combined with past anomaly patterns such that the combination is as close to the initial desired state as possible. From the results of rainfall forecasting for the three methods, the correlation and percentage error can be determined. It is discovered that the rainfall forecast during 15 May to 15 October 2010 in five regions by using the MCAM gave results that are similar to the observed stations at NKH, SUR, SAK, PCB, SPB, SRT, NST, and PHK1 which are satisfactory. The AM gave more correlation than the CAM and MCAM. However, the MCAM gave the minimum percentage error (13.66%), which shows that the rainfall forecast is closest to the actual observed value. The results are very similar to the actual data. Therefore, the MCAM is an alternative approach to forecast daily precipitation.

## Figures and Tables

**Figure 16:**
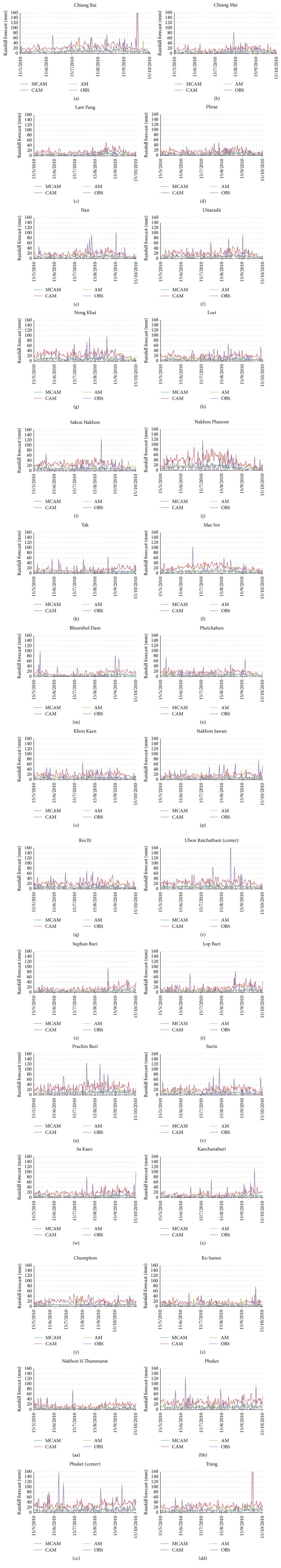
Time (day) during 15 May to 15 October 2010.

**Figure 17:**
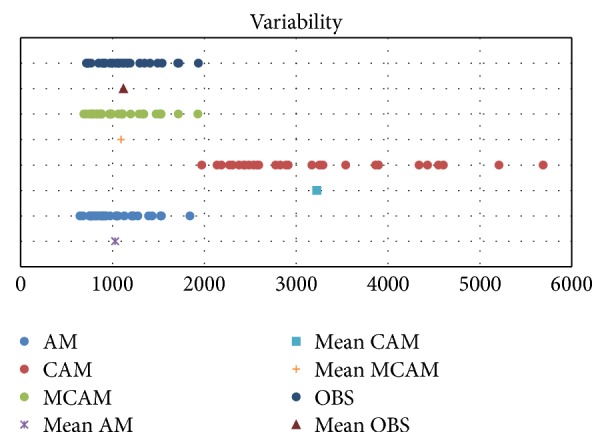
The variability plot from AM, CAM, and MCAM.

**Table 1:** Four variable predictors from the CFSR and CFSv2-Interim forecast dataset.

Predictors	Physical meaning	Units
T850	Temperature at 850 hPa height	°C
Q850	Moisture at 850 hPa height	g kg^−1^
G850	Geopotential height at 850 hPa	m
MSLP	Mean sea level pressure	hPa

**Table 2:** The locations of the thirty meteorological stations in the five regions in Thailand [27].

Number	Downscaling location	Station name	Lat. (°N)	Long. (°E)
1	North	Chiang Rai (CHR)	19.96	99.88
2		Chiang Mai (CHM)	18.79	98.98
3		Lam Pang (LPG)	18.28	99.52
4		Phrae (PHR)	18.17	100.17
5		Nan (NAN)	18.78	100.78

6	Northeast	Uttaradit (UTD)	17.62	100.10
7		Nong Khai (NKH)	17.87	102.72
8		Loei (LEI)	17.45	101.73
9		Sakon Nakhon (SNK)	17.15	104.13
10		Nakhon Phanom (NPN)	17.42	104.78
11		Tak (TAK1)	16.88	99.14
12		Mae Sot (TAK2)	16.66	98.55
13		Phumipol Dam (TAK3)	17.23	99.05

14	Central	Petchabun (PCB)	16.43	101.15
15		Khon Kaen (KHK)	16.46	102.79
16		Nakhon Sawan (NSW)	15.80	100.17
17		Roi Et (RET)	16.05	103.68
18		Ubon Ratchatani (UBT)	15.25	104.87
19		Suphan Buri (SPB)	14.47	100.14
20		Lop Buri (LBR)	14.80	100.62

21	Western	Pachin Buri (PBR)	14.05	101.37
22		Surin (SUR)	14.88	103.50
23		Sa Kaeo (SAK)	13.79	102.03
24		Kanchanaburi (KCN)	14.02	99.54

25	South	Chumphon (CHP)	10.48	99.18
26		Ko Samui (SRT)	9.47	100.05
27		Nakhon Srithummarat (NST)	8.54	99.95
28		Phuket (PHK1)	7.88	98.40
29		Phuket (Center) (PHK2)	8.15	98.31
30		Trang (TRG)	7.52	99.62

**Table 3:** Levels of correlation [31].

Value of *r*	Levels of correlation
0.90–1.00	Very high
0.70–0.90	High
0.50–0.70	Medium
0.30–0.50	Low
0.00–0.30	Very low

**Table 4:** Experiment cases for AM, CAM, and MCAM.

Method	Forecast field (from CFSv2)	Analysis field (from CFSR)	Hour (UTC)	Data availability
AM CAM MCAM	15 May to 15 October 2010	15 May to 15 October 1979–2009	0000, 0600, 1200, 1800	1979–2010

**Table 5:** Correlation between the observed rainfall and the predictors using AM and CAM taking into account the atmospheric predictors over Thailand regions including mean sea level pressure (MSLP), temperature (T850), moisture (Q850), and geopotential height (G850) at 850 hPa. Forecasts are for the months during 15 May to 15 October 2010 at the 30 TMD stations in Thailand.

Case	Station name	Rainfall forecast (mm) at 0000, 0600, 1200, and 1800 UTC								Rainfall (observed) (mm)
		G850		MSLP		Q850		T850		
		AM	CAM	AM	CAM	AM	CAM	AM	CAM	
1	CHR	1100.21	3451.80	1386.52	3993.94	1191.26	3626.82	1252.92	6646.73	1540.60
2	CHM	764.36	2149.88	1051.95	2610.86	896.22	2547.06	717.03	2421.42	973.00
3	LPG	640.02	1983.04	923.04	2428.05	823.71	2400.67	646.82	2287.80	725.50
4	PHR	800.46	2126.14	950.30	2603.08	913.97	2619.86	615.61	2407.90	900.50
5	NAN	788.54	2449.23	1042.01	2875.46	994.99	3027.15	779.32	2757.54	1297.30
6	UTD	915.98	2690.55	1297.06	3398.18	1068.90	3451.44	965.66	3147.13	1037.90
7	NKH	1066.28	3526.69	1366.87	3992.06	1424.60	4038.05	1246.67	4025.62	1409.00
8	LEI	749.12	2389.19	1110.56	3131.47	991.48	2927.04	868.20	2858.52	1160.40
9	SNK	1132.05	3690.38	1184.40	3861.29	1259.88	3969.75	1259.98	4054.12	1193.10
10	NPN	1888.09	5609.90	1495.63	5184.75	2071.19	5914.05	1920.34	6047.06	1710.70
11	TAK1	627.61	1912.34	723.37	2299.42	763.20	2180.71	604.57	2158.50	849.40
12	TAK2	1095.21	3532.98	997.92	3302.25	1405.34	3882.52	1019.35	3437.93	1081.00
13	TAK3	643.77	1885.03	622.83	2065.72	747.53	1881.22	571.52	2055.55	920.30
14	PCB	675.06	2217.09	930.55	2594.79	881.11	2754.29	821.04	2584.30	915.10
15	KHK	745.22	2499.90	1150.43	3110.14	957.19	3067.51	825.50	2976.56	996.70
16	NSW	711.83	2560.36	1038.69	2741.27	860.65	2477.84	894.80	2593.23	1167.70
17	RET	971.13	3009.09	903.08	3198.32	950.46	3482.88	1069.96	3475.87	996.60
18	UBT	1165.30	3893.10	1030.35	3593.99	1346.01	3902.88	1316.49	4068.83	1348.60
19	SPB	715.26	2229.01	845.54	2372.97	731.96	2213.57	767.56	2411.19	767.10
20	LBR	764.14	2384.77	728.29	2509.36	770.49	2797.93	772.85	2617.86	1114.80
21	PBR	1307.79	4313.54	1305.86	4235.60	1446.21	4107.28	1517.68	4697.70	1722.20
22	SUR	1021.78	3211.57	1044.88	3156.61	980.91	3250.48	1130.39	3438.41	1059.30
23	SAK	890.54	2768.69	777.12	2813.71	883.73	2934.10	1014.80	3045.63	1065.80
24	KCN	550.79	2055.37	917.29	2322.79	797.60	2084.03	731.27	2287.00	889.20
25	CHP	1015.86	3049.51	859.39	2978.16	1111.61	3369.49	1167.65	3620.65	739.70
26	SRT	661.61	2151.89	826.35	2426.40	852.59	2631.90	843.07	2729.10	733.60
27	NST	723.79	2080.71	891.57	2413.80	707.64	2676.97	788.80	2339.50	716.60
28	PHK1	1637.73	4639.42	1317.49	4287.73	1586.40	4564.23	1556.43	4699.62	1494.00
29	PHK2	1578.53	4620.26	1516.64	4573.52	1524.71	4458.55	1511.05	4755.84	1935.70
30	TRG	1292.68	3702.66	1351.59	3733.78	1789.55	9174.46	1293.73	4219.66	1124.60

Correlation		0.80	0.83	0.79	0.87	0.75	0.49	0.79	0.79	
*Average sum of rainfall (mm)*		954.69	2959.47	1052.92	3160.32	1091.04	3413.82	1016.37	3362.23	1119.53

**Table 6:** Correlation between observed rainfall from predictors by using the MCAM taking into account the atmospheric predictors over Thailand regions including mean sea level pressure (MSLP), temperature (T850), moisture (Q850), and geopotential height (G850) at 850 hPa. Forecasts are for the months during 15 May to 15 October 2010 at the 30 TMD stations in Thailand.

Case	Station name	Rainfall forecast (mm) at 0000, 0600, 1200, and 1800 UTC				Rainfall (observed) (mm)
		G850	MSLP	Q850	T850	
		MCAM				
1	CHR	1152.58	1341.63	1390.64	2222.82	1540.60
2	CHM	723.58	862.40	942.57	818.44	973.00
3	LPG	667.36	804.75	938.11	788.37	725.50
4	PHR	734.34	870.95	1033.62	846.44	900.50
5	NAN	820.41	959.24	1222.31	946.86	1297.30
6	UTD	938.87	1144.53	1250.46	1105.18	1037.90
7	NKH	1224.59	1341.58	1425.12	1379.49	1409.00
8	LEI	822.28	1030.72	1053.69	978.46	1160.40
9	SNK	1240.49	1333.80	1384.30	1345.27	1193.10
10	NPN	1906.72	1832.11	1968.16	2010.12	1710.70
11	TAK1	653.16	785.28	795.61	761.54	849.40
12	TAK2	1179.41	1156.93	1284.27	1175.31	1081.00
13	TAK3	637.91	695.94	721.82	699.15	920.30
14	PCB	755.31	885.35	1008.43	883.82	915.10
15	KHK	845.48	1024.82	1058.59	1037.39	996.70
16	NSW	882.81	915.06	829.23	872.11	1167.70
17	RET	1011.71	1102.13	1136.17	1181.14	996.60
18	UBT	1314.67	1232.44	1258.11	1352.00	1348.60
19	SPB	766.24	783.16	727.32	819.29	767.10
20	LBR	788.67	829.28	992.68	893.92	1114.80
21	PBR	1470.92	1445.15	1403.02	1581.46	1722.20
22	SUR	1062.12	1067.91	1103.94	1165.84	1059.30
23	SAK	903.97	964.35	960.12	1034.75	1065.80
24	KCN	663.69	750.81	665.12	776.28	889.20
25	CHP	1005.73	1021.47	1039.10	1196.53	739.70
26	SRT	720.20	812.05	889.38	897.08	733.60
27	NST	695.21	786.75	895.67	756.17	716.60
28	PHK1	1543.50	1454.38	1573.16	1548.05	1494.00
29	PHK2	1538.32	1527.25	1420.82	1547.63	1935.70
30	TRG	1231.64	1264.99	2997.66	1372.56	1124.60

Correlation		0.84	0.86	0.51	0.79	
*Average sum of rainfall (mm)*		996.73	1067.57	1178.97	1133.12	1119.53

**Table 7:** Correlation between observed and average rainfall from all predictors by average AM, average CAM, and average MCAM. For forecast predictor during 15 May to 15 October 2010 at 30 stations in Thailand.

Case	Station name	Rainfall forecast (mm)			Rainfall (observed) (mm)	Percentage error (%)		
		Average AM	Average CMA	Average MCAM		AM	CMA	MCAM
1	CHR	1232.73	4429.82	1526.92	1540.60	19.98	187.54	0.89
2	CHM	857.39	2432.30	836.75	973.00	11.88	149.98	14.00
3	LPG	758.40	2274.89	799.65	725.50	4.53	213.56	10.22
4	PHR	820.08	2439.24	871.34	900.50	8.93	170.88	3.24
5	NAN	901.21	2777.35	987.21	1297.30	30.53	114.09	23.90
6	UTD	1061.90	3171.83	1109.76	1037.90	2.31	205.60	6.92
7	NKH	1276.10	3895.61	1342.69	1409.00	9.43	176.48	4.71
8	LEI	929.84	2826.55	971.29	1160.40	19.87	143.58	16.30
9	SNK	1209.08	3893.89	1325.97	1193.10	1.34	226.37	11.14
10	NPN	1843.81	5688.94	1929.28	1710.70	7.78	232.55	12.78
11	TAK1	679.69	2137.74	748.90	849.40	19.98	151.68	11.83
12	TAK2	1129.46	3538.92	1198.98	1081.00	4.48	227.37	10.91
13	TAK3	646.41	1971.88	688.70	920.30	29.76	114.26	25.17
14	PCB	826.94	2537.62	883.23	915.10	9.63	177.31	3.48
15	KHK	919.59	2913.53	991.57	996.70	7.74	192.32	0.51
16	NSW	876.49	2593.17	874.80	1167.70	24.94	122.08	25.08
17	RET	973.66	3291.54	1107.79	996.60	2.30	230.28	11.16
18	UBT	1214.54	3864.70	1289.31	1348.60	9.94	186.57	4.40
19	SPB	765.08	2306.68	774.00	767.10	0.26	200.70	0.90
20	LBR	758.94	2577.48	876.14	1114.80	31.92	131.21	21.41
21	PBR	1394.38	4338.53	1475.14	1722.20	19.03	151.92	14.35
22	SUR	1044.49	3264.27	1099.95	1059.30	1.40	208.15	3.84
23	SAK	891.55	2890.53	965.80	1065.80	16.35	171.21	9.38
24	KCN	749.24	2187.30	713.98	889.20	15.74	145.99	19.71
25	CHP	1038.63	3254.45	1065.71	739.70	40.41	339.97	44.07
26	SRT	795.90	2484.82	829.68	733.60	8.49	238.72	13.10
27	NST	777.95	2377.75	783.45	716.60	8.56	231.81	9.33
28	PHK1	1524.51	4547.75	1529.77	1494.00	2.04	204.40	2.39
29	PHK2	1532.73	4602.04	1508.51	1935.70	20.82	137.75	22.07
30	TRG	1431.89	5207.64	1716.71	1124.60	27.32	363.07	52.65

*Correlation *(*r*)		0.82	0.79	0.80				
*R* ^2^		0.67	0.61	0.63				
*Average sum of rainfall (mm)*		1028.75	3223.96	1094.10	1119.53			
*RMSE*		202.00	2230.85	202.25				
*MAPE*						13.92%	191.58%	13.66%

## Appendix

### A. Time (Day) during 15 May to 15 October 2010

The time-series graphs showing the comparison between the amount of forecasted rainfall by using the MCAM, CAM, and AM and the actual observed rainfall (OBS) for all 30 stations in Thailand are shown in Figure 16.

### B. The Variability Plot from AM, CAM, and MCAM

The variability plot from these three approaches is shown in Figure 17.
